# Agentic AI as a coordination paradigm in digital health and agri-food systems

**DOI:** 10.1016/j.patter.2026.101496

**Published:** 2026-03-13

**Authors:** Anand K. Gavai, Miranda P.M. Meuwissen

**Affiliations:** 1 Industrial Engineering & Business Information Systems, University of Twente, Enschede, the Netherlands; 2 Business Economics Group, Wageningen University and Research, Wageningen, the Netherlands

**Keywords:** agentic artificial intelligence, personalized nutrition, metabolic health, health informatics, digital health equity, greenhouses, sustainable development goals

## Abstract

Digital health and agri-food data systems increasingly rely on sophisticated machine learning and data-sharing infrastructures. Yet persistent challenges in scalability, accountability, and public trust indicate that technical capability alone does not resolve systemic failure. This perspective argues that these limitations primarily arise from architectural misalignment with governance rather than from algorithmic insufficiency. Through a comparative examination of federated learning, blockchain-based infrastructures, and FAIR-aligned platforms, recurring coordination bottlenecks are identified across both health and agricultural domains. Building on these observations, this perspective introduces an agentic coordination model in which task-bounded agentic components operate under explicit institutional and regulatory constraints. The model context protocol (MCP) is presented as a reference mechanism for mediating policy, provenance, and accountability across distributed agents without centralizing control. Rather than prescribing a universal solution, this work frames agentic architectures as a governance-aware design space for future digital health and food systems.

## The bigger picture

Digital health technologies promise more personalized care, improved prevention, and better population health. Yet many digital health systems struggle to scale or to earn public trust despite rapid advances in artificial intelligence and data analytics. A central reason is that these infrastructures are often designed as technical solutions to problems that are fundamentally institutional. They depend on governance arrangements, regulatory compliance, and social consent elements that are rarely embedded into system design from the outset. As a result, interoperability remains fragile, accountability unclear, and public confidence difficult to sustain.

These challenges are not unique to healthcare. Similar barriers recur in other data-intensive domains, such as greenhouse agriculture and environmental monitoring, where large volumes of valuable data are generated continuously but remain difficult to reuse. Across sectors, fragmented governance, proprietary platforms, and ambiguous responsibility structures limit transparency and data sharing even when technical capabilities exist. By comparing digital health and agri-food data infrastructures, this perspective shows why technical innovation alone cannot overcome these constraints. Reimagining digital infrastructures as institutional systems where governance, ethics, and interoperability are treated as first-class design concerns opens new possibilities for trustworthy, cross-domain data use. Such an approach is essential not only for advancing digital health but also for addressing broader societal challenges at the intersection of health, food systems, and environmental sustainability.

## Teaser

Digital health systems increasingly rely on AI and large-scale data integration, yet their expansion is constrained more by governance and trust than by technical limits. In this perspective, the authors compare digital health and greenhouse data infrastructures to show how ethical, regulatory, and institutional barriers recur across domains and argue for agentic, privacy-preserving architectures that embed governance and interoperability by design.

## Introduction

Digital infrastructures in health and agriculture are undergoing rapid transformation, driven by advances in artificial intelligence, distributed data architectures, and large-scale analytics. Despite these advances, many deployed systems continue to struggle with interoperability, reproducibility, regulatory alignment, and institutional trust. These challenges persist even as technical performance improves, suggesting that architectural design choices rather than algorithmic limitations play a decisive role in shaping system outcomes.^1^^,^^2^^,^^3^ Similar coordination, accountability, and trust failures have been documented across digital health infrastructures,^4^ public sector data platforms, and agricultural data ecosystems,^5^^,^^6^ indicating that these challenges are systemic rather than domain specific.

In practice, coordination failures across digital health and agri-food systems rarely arise from algorithmic limitations alone. They often reflect layered institutional constraints that manifest at the level of decision-making: a system may “not be able” to act due to technical limitations, “lack the resources” to act due to administrative capacity, “decline to act” due to political or organizational priorities, or “be prohibited from acting” due to ethical or legal obligations. These distinct yet interacting constraints help explain why data-driven interventions frequently stall despite technical feasibility and why coordination, rather than optimization, emerges as the dominant challenge.

Across domains, similar patterns recur, as evidenced by persistent barriers in clinical data sharing,^7^ agricultural data governance, and digital infrastructure development.^4^^,^^8^^,^^9^ Health data platforms face difficulties in coordinating across institutional boundaries, while agri-food systems encounter comparable barriers in data sharing, provenance, and accountability. In both contexts, governance obligations are frequently addressed downstream, through audits or compliance layers, rather than embedded into system architecture. This separation between technical execution and institutional oversight has contributed to fragmented responsibility and limited scalability.

This perspective examines these challenges through a governance-oriented lens, focusing on how architectural paradigms shape coordination rather than on task-level performance. By comparing dominant approaches and synthesizing their limitations, the article motivates a shift toward architectures that explicitly integrate institutional constraints into system design.

This perspective proceeds in three steps. First, it analyzes prevailing digital data architectures through a comparative governance lens. Second, it synthesizes recurring coordination failures observed across health and agricultural systems. Third, it introduces an agentic coordination framework, instantiated through the model context protocol (MCP), as a conceptual response to these systemic constraints. Rather than proposing a new technical standard, this perspective examines how architectural design choices condition governance outcomes and uses this analysis to motivate a coordination-oriented alternative.

## Conceptual comparison of architectural paradigms

**Figure 1:**
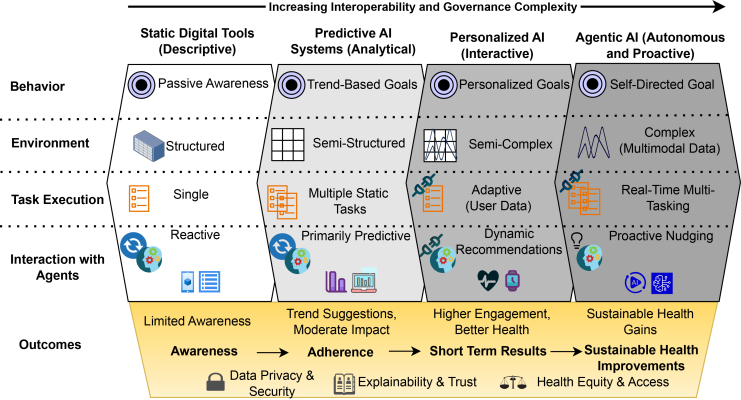
Evolution of digital health architectures and governance capabilities Conceptual evolution of digital data architectures in health and agri-food systems, highlighting shifts in coordination, governance, and institutional responsibility across centralized, federated, and interoperability-focused paradigms.

Figure 1 presents a conceptual comparison of three dominant architectural paradigms, federated learning,^10^ blockchain-based infrastructures,^11^ and FAIR-aligned data platforms,^12^^,^^13^ across governance-relevant dimensions. The comparison emphasizes structural trade-offs rather than empirical performance, highlighting how design assumptions influence accountability, coordination, and institutional alignment.

Federated learning emphasizes decentralized computation and privacy preservation yet often relies on implicit trust in coordinating entities and opaque aggregation processes. Blockchain-based infrastructures foreground immutability and transparency but can struggle with institutional adaptability and governance evolution. FAIR-aligned platforms promote interoperability and reuse but frequently depend on voluntary compliance and external enforcement mechanisms. Empirical studies in both healthcare and agri-food contexts show that federated learning^14^ reduces data centralization while introducing new challenges related to privacy leakage,^15^ coordination overhead, and trust in aggregation mechanisms.^16^ Although blockchain technologies are frequently proposed for traceability and provenance in healthcare and food supply chains, their governance implications, scalability constraints, and institutional adaptability remain contested.^17^^,^^18^^,^^19^ Evidence from biomedical research, omics data sharing, and agri-food systems highlights both the benefits and practical limitations of FAIR adoption, particularly regarding metadata quality, incentives, and institutional enforcement.^12^^,^^20^^,^^21^^,^^22^

Viewed collectively, these paradigms demonstrate that governance challenges are not incidental implementation issues but rather emerge from foundational architectural choices. Figure 1 therefore serves as a qualitative reference point for understanding how technical design decisions shape institutional outcomes rather than as a benchmark for system performance.

## Definition of agentic architectures

In this work, the term “agentic” refers to software entities capable of performing delegated tasks within explicitly bounded goals, operating under predefined institutional, regulatory, and contextual constraints. Unlike autonomous systems, agentic architectures do not imply independent goal formation or self-directed optimization. Instead, agency is limited, inspectable, and contingent on external policy, provenance, and oversight mechanisms.^23^^,^^24^

This framing emphasizes coordination and accountability rather than autonomy. Agentic systems are positioned as intermediaries that execute constrained actions within governance-aware environments rather than as independent decision-makers. Such an interpretation aligns agency with institutional responsibility and enables the embedding of governance requirements directly into system operation.

## Analytical framework

The analytical framework adopted here is comparative and conceptual, drawing on peer-reviewed literature, policy documents, and documented system deployments. Rather than evaluating technical performance metrics, the analysis focuses on how architectural paradigms structure coordination, accountability, and institutional alignment across domains.

The framework examines how design assumptions, such as decentralization, immutability, or interoperability, interact with governance requirements in practice. By applying a consistent set of governance-oriented criteria, the analysis surfaces cross-domain patterns that may not be apparent when systems are evaluated solely within their original application contexts. This governance-oriented perspective builds on institutional theories of digital infrastructure and data governance that emphasize coordination work, accountability structures, and socio-technical alignment.^1^^,^^23^

## Comparative analysis of existing paradigms

**Figure 2:**
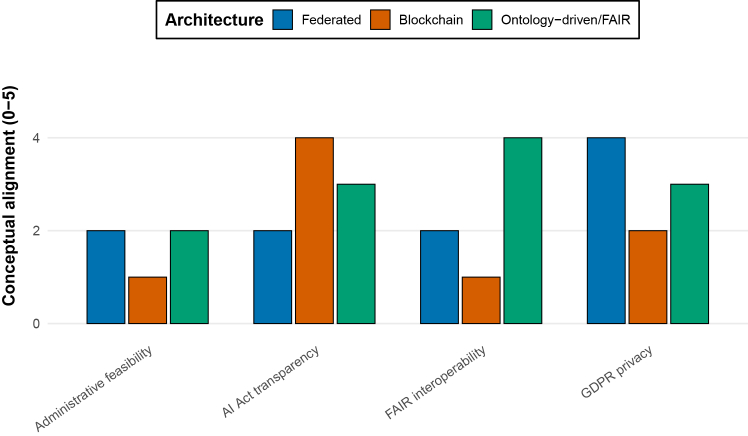
Relative alignment of digital architectures with GDPR, FAIR, and the EU AI Act Qualitative comparison of dominant architectural paradigms across governance dimensions. The schematic scores illustrate relative trade-offs discussed in the literature and are intended for conceptual comparison rather than quantitative evaluation.

Across paradigms, recurring limitations emerge. Mechanisms designed to protect privacy or ensure transparency often introduce new coordination burdens, while systems optimized for technical performance may obscure responsibility and accountability.^25^^,^^26^ Such trade-offs have been repeatedly observed in federated and distributed systems, where technical safeguards interact with organizational complexity and governance capacity.^4^^,^^27^ These patterns appear consistently across health^28^ and agricultural contexts,^29^ suggesting that they reflect structural constraints rather than domain-specific failures. Figure 2 contrasts dominant architectural paradigms along governance-relevant dimensions discussed in the literature.

## Governance refusals and design boundaries

The analysis reveals a set of governance-related refusals that shape the proposed architectural direction. These include refusals to centralize authority, to automate normative judgment, and to substitute technical compliance for institutional responsibility. Such refusals are not limitations of implementation but intentional design constraints. These design boundaries align with data protection principles articulated in the General Data Protection Regulation (GDPR), particularly regarding purpose limitation, accountability, and the avoidance of unjustified centralization of personal data.^30^ Similarly, emerging requirements under the European Union Artificial Intelligence Act (EU AI Act) emphasize human oversight and context-sensitive risk management, reinforcing the refusal to automate normative judgment within agentic coordination architectures.^31^ Beyond formal regulation, recent scholarship has highlighted the need for complementary audit ecosystems involving technical, institutional, and civil society actors, suggesting that architectural accountability cannot be reduced to statutory compliance alone.^32^

By articulating what the architecture does not attempt to do, these boundaries clarify the conditions under which coordination can remain accountable. They also motivate the transition toward an agentic coordination model that embeds governance considerations directly into system operation rather than addressing them post hoc. These architectural refusals resonate with regulatory and governance debates surrounding data protection, trustworthy AI oversight, and the growing recognition that accountability requires complementary audit ecosystems beyond statutory compliance.^32^^,^^33^

## Agentic coordination and the MCP

Building on the identified limitations, this work proposes an agentic coordination model in which agentic components operate within explicitly defined policy and provenance contexts. In this work, the term “agentic” refers specifically to agentic AI systems-computational components capable of goal-directed action under explicit contextual, institutional, and governance constraints rather than to generic software agents or unconstrained autonomous systems. The MCP^34^ is introduced as a reference mechanism for mediating interactions among agents, data resources, and governance constraints.^35^ The feasibility of such coordination depends on shared semantic infrastructures, including ontologies and controlled vocabularies, which play a critical role in interoperability across health and life science domains.^36^^,^^37^

Rather than functioning as a centralized authority, the MCP structures how contextual information, such as policy rules, data lineage, and accountability requirements, is made available to agents at execution time. This approach enables distributed coordination while preserving institutional oversight and traceability.^35^

The adoption of MCP-like mechanisms introduces non-trivial operational considerations. Policy formalization, ontology maintenance,^38^ and agent certification require sustained institutional investment. Without shared infrastructure or intermediaries, these requirements may disadvantage smaller organizations or resource-constrained settings, highlighting the importance of governance support structures alongside technical design.

## Proposed reference architectures

**Figure 3:**
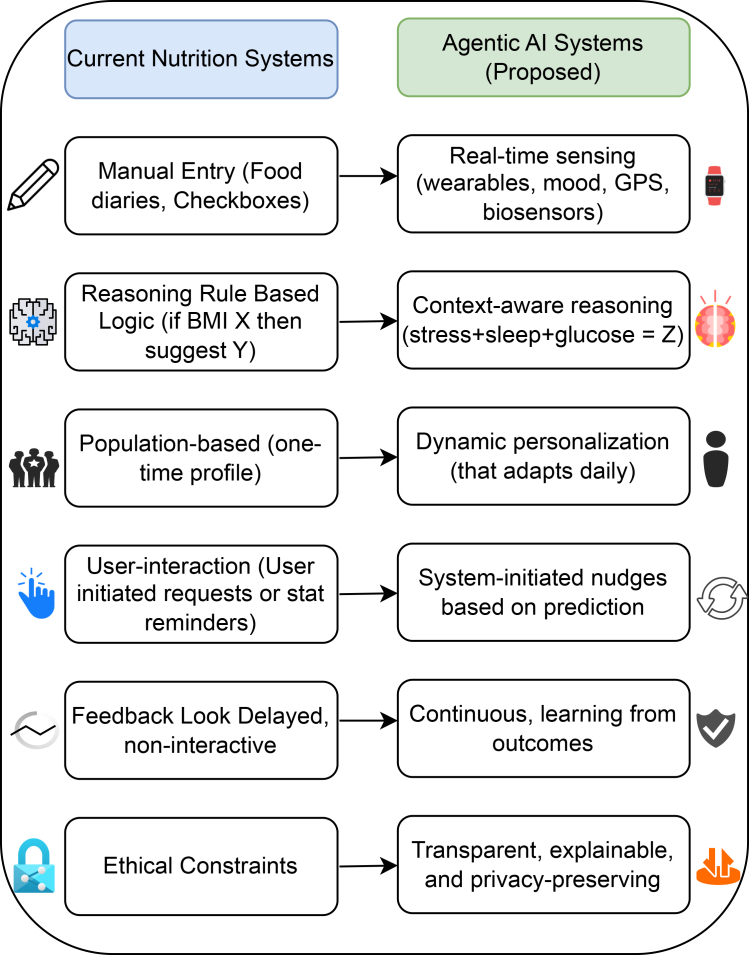
From personalized nutrition pipelines to context-aware agentic coordination Proposed reference architecture for agentic coordination, illustrating how agentic components interact with policy, provenance, and data services within a governance-aware environment.

**Figure 4:**
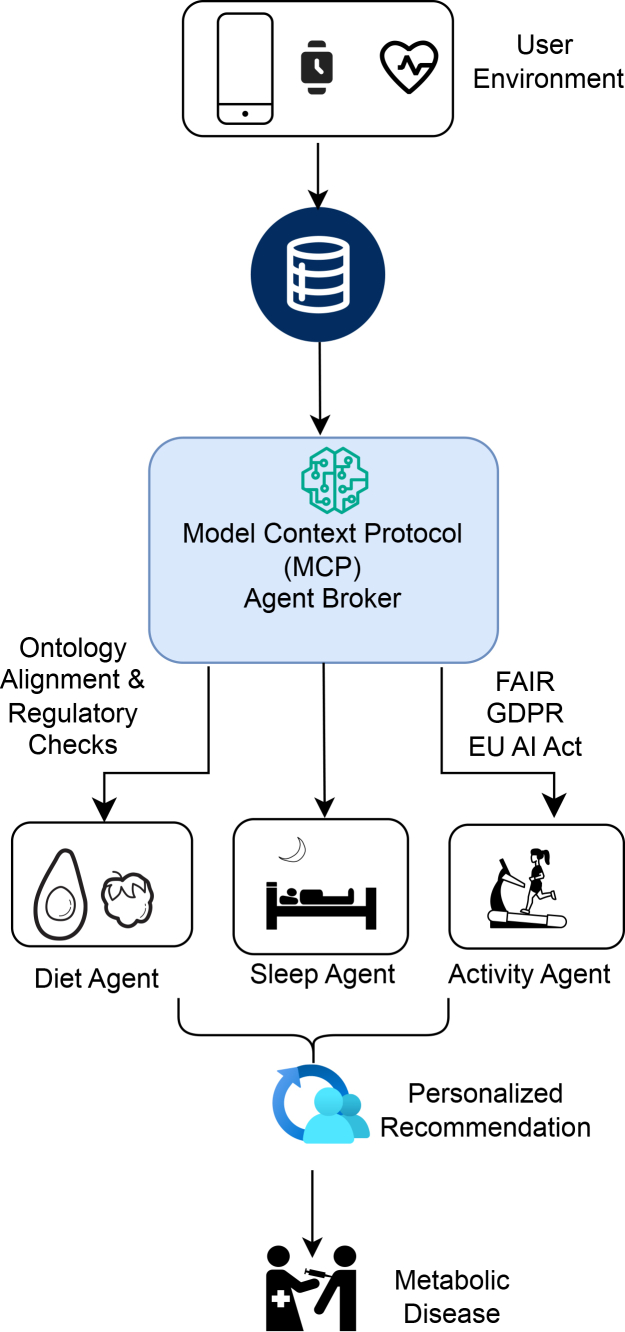
Governance-aware multi-agent coordination using the model context protocol Alternative reference configuration demonstrating agentic coordination across institutional boundaries, emphasizing distributed control and contextual policy mediation.

Figures 3 and 4 illustrate proposed reference architectures that instantiate agentic coordination within governance-aware environments. These diagrams depict conceptual deployment patterns rather than observed systems, emphasizing structural relationships among agents, policy services, and data resources. Figure 3 illustrates agentic coordination within a single institutional context, while Figure 4 extends the same coordination logic across institutional boundaries, highlighting differences in governance scope rather than architectural mechanism.

The reference architectures are intended to support discussion of coordination logic and institutional integration rather than prescribe specific implementation choices. They demonstrate how governance constraints can be made operational through architectural design without centralizing control.

## Discussion

The findings presented here suggest that reproducibility, accountability, and trust in digital health and agricultural systems emerge less from algorithmic sophistication than from institutional alignment. Architectural choices that separate technical execution from governance oversight risk reproducing coordination failures even as computational capability improves.

Agentic coordination reframes reproducibility as a governance property rather than a purely technical one, emphasizing traceability, policy context, and institutional responsibility. This shift raises ethical considerations^39^ related to collective consent, shared stewardship,^23^ and the distribution of administrative burden across stakeholders.^33^ Prior work in biomedical ethics and data governance underscores that collective consent, stewardship models, and auditability are essential complements to technical design in sustaining public trust.^40^

Rather than resolving these tensions, the proposed framework makes them explicit, enabling more deliberate institutional negotiation. In doing so, it positions governance-aware architecture as a prerequisite for sustainable digital infrastructure rather than as an optional compliance layer. Taken together, these observations point toward practical pathways for building open, reproducible, and trustworthy digital infrastructures across food and health systems.

This perspective argues that persistent failures in digital health and agri-food data systems reflect architectural misalignment with governance rather than insufficient technical capability. Examining dominant paradigms through a comparative lens highlights the need for coordination mechanisms that embed institutional constraints directly into system design.

Agentic coordination frameworks, exemplified by the MCP, offer a conceptual pathway toward governance-aware digital infrastructure. Their evaluation should prioritize accountability, traceability, and institutional fit over predictive performance alone. Regulatory sandboxes and shared public infrastructure may provide important venues for testing such approaches under real-world conditions.

## Acknowledgments

This work was supported through the 'High Tech for a Sustainable Future' capacity building programme of the 4TU Federation in the Netherlands.

## Declaration of interests

The authors declare no competing interests.

## Declaration of generative AI and AI-assisted technologies in the writing process

During the preparation of this manuscript, generative artificial intelligence (AI) tools were used to improve the narrative flow, enhance clarity, and make the content more accessible to a broad audience. The authors take full responsibility for the integrity, accuracy, and content of the publication.
