# Agentic AI as a coordination paradigm in digital health and agri-food systems

**DOI:** 10.1016/j.patter.2026.101584

**Published:** 2026-05-13

**Authors:** Anand K. Gavai, Miranda P.M. Meuwissen

## Main text

(Patterns *7*, 101496; March 13, 2026)

In the original version of this article, text in the section “conceptual comparison of architectural paradigms” did not fully align with the content of Figure 1 due to an error during manuscript revision. The first few sentences of this section have been revised to clarify this.

**Original text:** "Figure 1 presents a conceptual comparison of three dominant architectural paradigms, federated learning,^10^ blockchain-based infrastructures,^11^ and FAIR-aligned data platforms,^12,13^ across governance-relevant dimensions. The comparison emphasizes structural trade-offs rather than empirical performance, highlighting how design assumptions influence accountability, coordination, and institutional alignment."

**Corrected text:** "Figure 1 presents a conceptual illustration of the evolution of digital architecture. The comparison emphasizes structural trade-offs rather than empirical performance, highlighting how design assumptions influence accountability, coordination, and institutional alignment.

Within this broader architectural evolution, three dominant paradigms, federated learning,^10^ blockchain-based infrastructures,^11^ and FAIR-aligned data platforms,^12,13^ have emerged as key approaches to addressing governance and coordination challenges."

The article has now been corrected online. The authors apologize for any confusion this may have caused.

